# Where next for partial randomisation of research funding? The feasibility of RCTs and alternatives

**DOI:** 10.12688/wellcomeopenres.19565.1

**Published:** 2023-07-17

**Authors:** Tom Stafford, Ines Rombach, Dan Hind, Bilal Mateen, Helen Buckley Woods, Munya Dimario, James Wilsdon

**Affiliations:** 1 The University of Sheffield, Sheffield, England, UK; 2 Wellcome Trust, London, England, UK; 3 Research on Research Institute, London, England, UK; 4 University College London, London, England, UK

**Keywords:** metascience, metaresearch, review, experiments, lottery

## Abstract

We outline essential considerations for any study of partial randomisation of research funding, and consider scenarios in which randomised controlled trials (RCTs) would be feasible and appropriate. We highlight the interdependence of target outcomes, sample availability and statistical power for determining the cost and feasibility of a trial. For many choices of target outcome, RCTs may be less practical and more expensive than they at first appear (in large part due to issues pertaining to sample size and statistical power). As such, we briefly discuss alternatives to RCTs. It is worth noting that many of the considerations relevant to experiments on partial randomisation may also apply to other potential experiments on funding processes (as described in The Experimental Research Funder’s Handbook. RoRI, June 2022).

## Introduction

In recent years, applications of partial randomisation to research funding processes have received growing attention from research funders, meta-researchers and the wider research community ( Nature editorial, 2022; Woods & Wilsdon, 2021a). Partial randomisation, also known as focal randomisation or random selection, is a method for allocating research funding. It is used in addition to peer review, where peer review has reached the limits of efficacy and fairness, and the comparative qualities of applications are largely indistinguishable or ‘equally good’ ( Bedessem, 2020). In its partial form, only a subset of applications are subject to random selection, once those which are evaluated as clearly fundable or clearly non-fundable have been removed.

A recent study ( Woods & Wilsdon, 2021b) found that the strongest motivator for funding institutions to use partial randomisation is fairness: a fairer decision-making process when peer review had reached its limits; fairer to applicants, as it is blind to institution, geographical location, race, gender, discipline and methodology; and also a transparent process and therefore easier to communicate and understand funding decisions. Other organisational motivators are the desire to break deadlocks in, or reduce time spent on panel decision making, and to ameliorate risk aversion or other concentrations of awards so as to facilitate the funding of a greater plurality of research topics and methodological types.

Pilots of steadily increasing volume and sophistication have been conducted ( Bendiscioli *et al.*, 2022). There are some clear emerging lessons, but also much that remains unknown. This includes the extent of any ultimate benefits in terms of reduction of biases or gains in efficiency, as well as assurance that harms, such as trust in and acceptance of funding allocation that involves partial randomisation, are acceptable. Strategies for enhancing the evidence based around partial randomisation could be divided into three general categories:

### a) “Steady as she goes”

Conduct more smaller scale pilots. Concerns regarding this approach are that another five years of small-scale pilots will not aggregate to a compelling evidence base. By the same logic of “the plural of anecdote is not data”, smaller, potentially flawed, studies never add up to the evidential power of a more comprehensive, systematic trial.

### b) “From model to implementation”

Some, e.g. Gross & Bergstrom (2019), have asserted that the costs to research time are sufficient to warrant major overhauls of research funding allocation processes, including partial randomisation. By this account, we have little to lose and much to gain by a larger scale implementation of partial randomisation. However, the concern is that abstract models under-estimate system complexity, particularly with respect to stakeholder aspects (see Barlösius & Philipps, 2022; Liu *et al.*, 2020), namely the perception of, and reaction to, of funding allocation by lottery among those who apply for funding and those who are awarded funding. Additionally, larger scale implementation assumes that the motivations for partial randomisation are agreed, something which is not clear ( Woods & Wilsdon, 2021a).

### c) Funder experiments

A third option, which is the focus of this paper, is to conduct larger scale experiments, across multiple funding agencies if necessary, to produce a compelling test of the benefits of partial randomisation. An obvious candidate method is a randomised controlled trial (RCT) of the effects of partial randomisations, honouring the “gold standard” of evidence for medical innovations.

### Outline of work

In this article we outline essential considerations for any study of partial randomisation of research funding, and consider scenarios in which RCTs would be relevant. We highlight the interdependence of target outcomes, sample availability and statistical power for determining the cost and feasibility of a trial. For many choices of target outcome RCTs may be less practical and more expensive than they first appear (in large part due to issues pertaining to sample size and statistical power). As such, we also introduce and briefly discuss alternatives to RCTs. It is worth noting that many of the considerations relevant to experiments on partial randomisation may also apply to other potential experiments on funding processes (see Bendiscioli *et al.*, 2022).

## Considerations for studies of the potential benefits of partial randomisation

Designing a robust study requires a clear understanding of the precise question that is being answered, more technically, the ‘treatment effect’ that one seeks to estimate. This ‘estimand’ is best described by its five constituent attributes: (i) the population of interest, (ii) the ‘treatment’ conditions compared, (iii) the outcome measure of interest, (iv) the population-level summary measure used to describe how outcomes differ under the aforementioned conditions (e.g., a risk ratio, odds ratio, etc.), and (v) acknowledgment and management of intercurrent events that can impact interpretation of the results (e.g., career change or securing funding from another source). For more detail see the *ICH E9 (R1) addendum on estimands and sensitivity analysis in clinical trials to the guideline on statistical principles for clinical trials* (2020)

### Defining the estimand for studies of partial randomisation of research funding

Below is a brief discussion of the nuances that need to be considered when defining the estimand in this context, and particularly those pertaining to outcome selection.

Focusing initially on describing the population of interest, most examples of partial randomisation that have been undertaken to date have focused on the applicants or reviewers associated with a specific scheme run by an individual funding organisation ( Woods & Wilsdon, 2021a; Woods & Wilsdon, 2021b). To assume that these results are generalisable to other funders and national research systems, even when there is overlap in archetypal applicants (e.g. schemes limited to early career researchers in a specific area of biomedical science), is probably not appropriate given the non-overlapping pools (whether geographic or otherwise) that funders receive applications from. Instead, to make a definitive claim would require a trial that by design did not limit its population of interest to an individual funding organisation.

Similarly, there are nuances to the operationalisation of the ‘partial randomisation’ concept (see Woods & Wilsdon, 2021a for details on pre-existing funder experiments), which in turn influence the nature of the treatment conditions being compared.

Finally, there are a number of different outcomes that can be measured which assess the potential benefits of partial randomisation. Critical to effective study design is the declaration, **in advance**, of the way the target outcome measure(s) will be operationalised. It is this prespecification which defines the study as a ‘Trial’, and is as important to successful inference interpretation as the ‘Randomised Control’ aspect of an RCT ( Simmons *et al.*, 2011). Candidate outcomes include those that measure impact on the funded portfolio, on diversity of applicants, or efficiency of review and/or decision process. Notably, these outcomes either do not have fully satisfactory outcome measures, or they afford a choice between different outcome measures which trade-off ease and accuracy. For example, the target outcome of enhanced diversity of applicants could be operationalised using the demographic parity (mathematical) definition of fairness (i.e., trying to ensure that a diversity of applicants in the funded portfolio reflects the application rate for each demographic group in the applicant pool).

In practice, this would be implemented by requesting applicant ethnicity data and comparing the impact of partial randomisation, and comparing that to the diversity in the portfolio funded under standard operating procedures. Importantly, any specific operationalisation will be limited by the principle on which it is based (i.e., there are many definitions of fairness, and satisfying several simultaneously can be impossible; Kleinberg *et al.*, 2016), what it does not ask (e.g. other dimensions of diversity such as sex or socioeconomic status) and how it asks (e.g. the categories for ethnicity which define how applicants are asked to report). In addition, both potential benefits and harms will need to be assessed by any outcome measure. Considerations of potential target outcomes and outcome measures are shown in Table 1.

**Table 1.** Possible outcomes: a non-exhaustive list.

Outcome	Issues
*Benefit outcomes*	
Fairness	Lack of a relatively objective criterion (gold standard) measure ( Brezis & Birukou, 2020). Operationalised as distributive fairness (fairness of outcomes), may require large numbers/long timescales, to spot differences in clustering of grants. Procedural / informational fairness would require researchers to observe and code committee work or an adjudication committee to assess content of rejection letters.
Efficiency: time to deliberation	Objective, continuous (therefore efficient) measure and has been used successfully ( Bendiscioli *et al.*, 2022)] but may not be seen as important to the public.
Efficiency: appeals	Objective, dichotomous measure. May require large sample sizes, depending on base rate. Not universally applicable and not every funder permits appeals.
Diversity	Requires operationalisation by applicant demographic (gender, ethnicity, etc.) or topic (academic disciplines and research methodologies). The latter might require coding manuals and coders / adjudication committees to resolve.
High-risk, high reward projects	Risk is subjective and would require researchers to observe and code applications or an adjudication committee. Reward would require long timescales
Exceptional scientific advances	Requires a long timescale and large numbers (very rare event). Would require researchers to observe and code applications or an adjudication committee running over years.
*Harm outcomes*	
Application quality	Requires coding manuals and coders / adjudication committees to resolve.
Questionable research practices	Subjective. Would require advertisement of the RCT between lottery and usual practice, as well as two researchers to code grant applications against a framework for QRP.
Reputational damage to funder	Not subject to experimental design. Can perhaps be operationalised via perceptions of individual scheme and/or individual scheme applicants.
Stigmatisation of awardee	Timescales likely to be undesirable. Measurement likely to be problematic.

In essence, partial randomisation of research funding represents a broad church of meta-research questions and associated approaches, and thus, there are many estimands that one might consider running a study to address. In the next section we explore how study design might be influenced by the choice of estimand. Regardless though, collaboration and coordination amongst funders to undertake any study which conforms to a set of standard estimand definition is a non-trivial endeavour, and this is where organisations such as RoRI can play a role in facilitating collaborations and exchange across different funders.

### How study design impacts partial randomisation meta-research

In partial randomisation research, the choice of outcome measures, particularly the primary outcome measure, plays an important role in determining the study design. Some outcomes may not require full implementation of allocation of awards via partial allocation (see “shadow experiments” below). On the other hand, if the outcomes of primary interest can only be measured post-funding selection, or even project completion, such as appeals against funding decisions, the impact of funding on career development, or the impact on scientific advancement, then an RCT may be the most suitable design option.

Once stakeholders have chosen a ‘primary outcome’ - the target outcome that key stakeholders (e.g., funders, researchers, funding panels, patients) would agree is the most important -, and a plausible operationalisation of it in an outcome measure, it is necessary to consider the target effect size. This is the difference between the two experimental conditions (e.g. partial randomisation and funder standard practice) which would be worth detecting if it existed.

The statistical power of a trial is the probability of detecting the target effect, should one exist. Given the time, cost and effort of RCTs this probability should be high (e.g., at least 90%, if not prevented by feasibility constraints). The target effect size, along with elements of trial design such as the sample size and number of conditions, define statistical power. All other things being equal, larger effects can be detected with smaller sample sizes.

The primary outcome also defines the unit of analysis. In medical trials, the unit of analysis is often patients. For our purposes it may be grant applicants, grant applications, peer reviewers, funder panellists, awarded grants or successful awardees. The questions being asked influence the outcome, the unit of analysis, and the nature of outcome assessment. Table 2 illustrates how different potential target outcomes affect trial practicality via determination of available sample sizes.

**Table 2.** Target outcome, unit of analysis and sample availability for one funding call.

**Target outcome**	applicant diversity, beliefs about partial randomisation	proposal novelty, ambition/risk	reviewer burden, review consistency	project productivity, diversity characteristics of awardees, awardee reaction to award by partial randomisation
**Unit of analysis**	APPLICANTS	APPLICATIONS	REVIEWS	AWARDS
**Sample available**	number of investigators	number of applications	number of applications x reviews per application	number of applications x proportion funded
**Illustrative numbers** assuming 100 applications, 3 investigators, 4 reviews per applications, and a 10% success rate	**300**	**100**	**400**	**10**

These considerations show that considerable range exists under the headline call to conduct RCTs of partial randomisation. Different choices of target outcome(s), and so of unit of analysis, have large implications for the ease, rate and cost of recruitment for an adequately powered trial. In addition, different target outcomes afford outcome measures which are more or less satisfactory in the terms of capturing the true value of the outcome and delay required to collect them (at one extreme being the target outcome of selecting for high-risk, high-value discovery-mode research. While obviously laudable, the delay between adjustments to any funding process and the outcome of increased rates of fundamental breakthroughs alone makes this a less practical target outcome).

### Indicative protocol

As a thought-experiment and to illustrate the aforementioned issues in more detail, we present an indicative protocol for a RCT of partial randomisation in the context of a major UK health research scheme, the National Institute for Health and Care Research (NIHR) Health Technology Assessment (HTA) programme.

**Research question:** Does partial randomisation enhance the impact of the funded portfolio?

**Population:** Research applications to the NIHR HTA programme.

**Intervention:** Receipt of funding will be allocated via lottery for proposals rated as fundable by external peer review, without going to panel discussion.

**Control:** “standard practice”, i.e., adjudication by committee discussion along funder specific guidelines.

**Outcome:** Patients benefit arising from both portfolios, calculated by the number treated with the HTA-approved products (assuming approved) × Quality Adjusted Life Years (QALY). Impact per £ can be calculated by dividing by total portfolio value.

**Logistics:**

Practicalities around recruiting and randomising study participants (i.e., grant applications) require careful consideration.

- How will grant applicants be informed that funding decisions may be based on partial randomisation? Would all the usual candidates be willing to participate in the trial, or apply for alternative funding elsewhere?
- Timing of randomisation: all funding applications would be required to pass some minimum quality standard, and would then be randomised to either be allocated funding via a lottery, or by the committee.
  When would be the most appropriate time to perform the randomisation, and who would do this?
  Online randomisation systems, with appropriate stratification or minimisation by funder, funding round and other important factors can easily be implemented by a clinical trials unit (CTU). Specific guidance would have to be drawn up to ensure delegates of the funders are able to perform the randomisation.
- Details on how grant applicants are informed of the outcomes need to be considered.

**Sample size:**

For the purposes of this example, assume that the allocation of research funding by lottery would be deemed successful if the total patient benefit was raised - on some assumed, continuous, outcome measure - by 0.4 of a standard deviation (actually a moderate, rather than small effect). Based on these expected event rates, using a two-sided 5% significance level and 90% power, a total sample of 264 funded studies (132 per arm) would be required. Enough studies would have to be randomised to either approach of funding allocation to result in the required number of funded studies, i.e., allowing for the proportion of studies not receiving funding. If the success rate is 20%, this means 660 applications would be required *in each arm* (1320 total applications). Larger effect sizes would decrease the required sample size, though might be less realistic.

**Feasibility:**

Finally, the total duration and cost of the trial can be estimated based on the recruitment rate. Let us imagine a funding panel that adjudicates on around 30 applications at each of three time points per year. Let us further imagine that 20/30 grants are neither clearly fundable nor clearly unfundable and that applications in this middle segment are randomised to lottery or usual practice. As the committee sits three times per year, a total of 60 grants might be entered into an RCT comparing randomisation lottery or usual practice. This number may be optimistic depending on: (1) how programme-level funding limits apply; or, (2) whether panels only make funding recommendations to a government department rather than directly allocate grant income themselves. Let us imagine this funder has three other panels, so during the course of one year all their funding panels will contribute 180 grants. Given these considerations, it would take (applicants * number of treatment arms) / applications per year years to allocate sufficient grants for an adequately powered RCT of partial randomisation for this target outcome and trial design from this large multi-panel funder alone. In our example this calculation is (660*2/180), which is 7.3 years for the allocation stage alone.

Following allocation we might assume 3–6 years from funding to publication. This *may* be followed by approval for wider use (e.g., by the National Institute for Health and Care Excellence [NICE] in the UK) and then commissioning by healthcare delivery agencies. A speedy estimate of these aspects is 10 years ( Morris *et al.*, 2011), which means that final outcome data for this trial would be available at approximately year 17 of its existence, at the earliest. Many, but not all, non-healthcare funding domains will have equally lengthy delays between research completion and feasible outcome measurement.

This makes clear the need for funder collaboration to support timely recruitment to trials, as well as - perhaps - to focus on outcome measures which afford early assessment. It also suggests that there may be benefits to explore alternatives to RCTs (see below).

**Typical costs:**

The average cost per participant in medical industry trials is around US$41,413 (or £29,744), based on FDA data from 2015 to 2017 ( Moore *et al.*, 2020). By comparison, the average cost per participant in NIHR HTA-funded trials is probably around £3000 per participant. An RCT comparing allocation by lottery versus usual funding panel practice would have grant applications, not humans as its ‘participants’. Depending on the outcomes of interest (some of which would require expensive data collection infrastructure) the trial could be relatively cheap, perhaps £1m.

Costs to run, whether supplied directly to a third-party to administer the trial or provided “in kind” by funding agency staff who administer the trial is separate from the funding allocation to grant awards. Funding allocated to awards by partial randomisation, while necessary for a trial, would be allocated anyway, even if the RCT was not conducted. The money will just be allocated differently because some of it will be allocated at random rather than by funding panel adjudication.

## Alternative to RCTs

There exist a number of alternative methods which are currently used where RCTs are less feasible, or inappropriate. We briefly review their strengths and weaknesses here.

### Causal inference methods

The baseline assumption of many researchers, and the impetus for RCTs, is that observational studies may not yield reliable inferences of treatment effects ( Young & Karr, 2011). Statistically controlling for confounders in observational data is challenging ( Westfall & Yarkoni, 2016). Moreover, natural assumptions, such as balanced ‘unmeasured’ covariates that arise from formal randomisation are more difficult to justify in observational settings, thus leaving the door open to residual confounding which can produce misleading/inaccurate results (unbeknownst to the analyst).

However, a new generation of causal inference methods have recently gained attention which purport to allow more reliable inference from observational data ( Pearl *et al.*, 2016). For example, Hernan & Robins ( 2016, see also Hernán *et al.*, 2016) propose guidelines for causal inference from large observational databases. Like an RCT, it is necessary to specify the population, intervention, comparator and outcome, as well as any important mediators or moderators, with these being used to attempt mitigation of selection biases. For an exemplar demonstration of how to compare these observational methods to an RCT see Lodi *et al.*, 2019. These approaches have proven robust in estimating average treatment effects, as demonstrated by Hernán and colleagues in their observational data-based confirmation of the effectiveness of the mRNA COVID-19 vaccine ( Dagan *et al.*, 2021). 

Caveats include that very large data sets are required, so the application of such methods would require data collection and harmonisation across multiple decades and funding agencies. Additionally, successful inference can only be done if the population of grant-schemes observed includes some occurrence of allocation by partial randomisation.

Finally, it should be humbling that studies by researchers at social media platforms with access to both billions of data points and the ability to run true experiments, akin to the RCTs discussed here, have shown that even the latest generation of causal inference methods may not be successful at accurately revealing the true causes of things ( Gordon *et al.*, 2019; Gordon *et al.*, 2022), or may have restricted applicability to novel phenomena ( Eckles & Bakshy, 2021).

### Qualitative comparative analysis

Qualitative Comparative Analysis (QCA; Marx *et al.*, 2014) is a formal method of studying causality in a simple data table of binary or ordinal variables from small-medium ‘N’ samples (8–200). The method uses Boolean algebra to understand the necessary or sufficient conditions for outcomes to occur. Methods such as QCA might be attractive if the numbers required by probabilistic causal inference approaches are deemed infeasible, due to the sample sizes required, or if funders want exploratory studies of the effects of different factors on outcomes.

### Natural experiments

A special case of causal inference with observational data is the existence of natural experiments ( Dunning, 2012). An example is regression discontinuity designs, which take advantage of arbitrary thresholds which divide cases near that threshold into two groups, despite being essentially similar. This analysis has been applied to study the effect of funding success on longer term research career outcomes ( Bol *et al.*, 2018; Wang *et al.*, 2019). It may be that there are similar natural experiments possible within the funding system.

### Shadow experiments via simulated outcomes

Outcome measures that can be assessed without implementing selection, such as diversity of awardees, or time taken to make funding decisions may not require the full process of randomisation and subsequent awards. Instead, the “as if” impact of partial randomisation can be simulated and the outcome compared directly with outcomes from the standard procedure. In other words, funders could choose to base their funding decisions on their usual decision making process while the experiment is ongoing, using standard procedure as data collection for a “shadow experiment” on partial randomisation.

This approach is statistically powerful, since the entire universe of outcomes which would be produced by partial randomisation can be simulated and used as a basis for comparison. An advantage of partial randomisation is that it is a process which can be easily modelled. Panel review is non-reducible, it exists because the selection of projects to fund made by a panel is not knowable in advance. In contrast, partial randomisation is a minimal process which could be applied to the population of grants under consideration by a panel, without implementing it as the selection process. So, for example, if a panel funded 10 grants from 100 fundable grants, it is possible to identify the precise statistical distribution awards which would have been made by partial randomisation.

## Conclusions

It is challenging to trial novel methods of funding allocation and evaluation. Peer networks of funders offer a route to sharing lessons from pilots and trials, and to building a more robust evidence base. There is more scope for funders to work together—including through the RoRI consortium—to deepen our shared understanding of the value and limitations of partial randomisation and other experimental methods.

There is a need for more robust experimental studies, with defined baselines and controls: ideally involving multiple funders, which will allow for comparison across funding systems. The potential of early pilots will not be realised without more rigorous, long-term experiments which can generate transferable evidence of the pros and cons, opportunities and limitations of specific interventions. Moving beyond analysis of changes to applicant and allocation processes to study the full impacts of different funding methods on research outcomes will require sustained analysis, as these will take several years to become apparent.

At the same time, a move towards formal RCTs in this arena is not straightforward. RCTs are complex design objects. A defining consideration is deciding in advance what target outcome you wish to focus on. This choice may render the necessary sample size for adequate statistical power, and related cost and ease of recruitment, prohibitive. Larger samples will need coordination between multiple funding agencies.

## Data Availability

No data are associated with this article.
