# Supplementary material for: Where next for partial randomisation of research funding? The feasibility of RCTs and alternatives
Source: Wellcome Open Res. 2024 May 30;8:309. Originally published 2023 Jul 17. [Version 2] doi: 10.12688/wellcomeopenres.19565.2 (PMC10474338; doi:10.12688/wellcomeopenres.19565.2)
Supplement: Peer review history

# Reviewer report: Reviewer response for version 2

**DOI:** 10.21956/wellcomeopenres.24789.r85279

**Published:** 2024-06-10

**Authors:** Merle Jacob

**Affiliations:** 1 Professor in Research Policy, Lund University, Lund, Sweden

I am happy with the revised version and recommend indexing.

Is the review written in accessible language?

Partly

Are all factual statements correct and adequately supported by citations?

Yes

Are the conclusions drawn appropriate in the context of the current research literature?

Partly

Is the topic of the review discussed comprehensively in the context of the current literature?

Yes

Reviewer Expertise:

Research funding, research policy

I confirm that I have read this submission and believe that I have an appropriate level of expertise to confirm that it is of an acceptable scientific standard.

# Reviewer report: Reviewer response for version 1

**DOI:** 10.21956/wellcomeopenres.21675.r65750

**Published:** 2023-09-04

**Authors:** Merle Jacob

**Affiliations:** 1 Professor in Research Policy, Lund University, Lund, Sweden

The paper addresses the problem of how to design a study to implement RCTs in research funding. This is a narrow area of research outside of the discourse about clinical trials so the literature is rather sparse. In my view the language of the paper may not be entirely accessible to the rest of the research community who work with research funding.

 The paper might also benefit from considering the fact that RCTs need to focus on funder objectives. Increasing the perception of procedural fairness? Reducing selection bias, etc. are all potential objectives. Some outcomes may be more or less amenable to being grouped together in the same trial. The current paper is not clear on which objectives it takes on board in its worked example or thought experiment.

 I agree that a proper experiment would require multiple funders but there are very few funding situations to which the results of this experiment would be useful. Real world cases with multiple funders are rare and often further complications such as different authority rights and jurisdictions arise.

 Perhaps considering applying the method to more than one funding instrument may be a potential alternative. 

 All research on funding faces a problem of selection bias. We can only select from those who apply and the character of the applicant sample can only be partially controlled for by qualification criteria. Who applies is therefore dependent not on instrument design and likewise who gets funded is only partially determined by the character of the selection process. Here the analogy with clinical trials is only partially helpful because the sample of patients who are potential candidates is non random in the sense that they must be selected from those afflicted. With funding, not all those eligible are likely to apply and those who apply may not represent a random sample of all the characteristics of the set of potential applicants. 

 Generally, the paper is though provoking and I could comment reams on this but the key issue here is that RCTs are a solution in search of a problem. If the problem can be identified then we can better discuss the efficacy of RCT as a solution.

Is the review written in accessible language?

Partly

Are all factual statements correct and adequately supported by citations?

Yes

Are the conclusions drawn appropriate in the context of the current research literature?

Partly

Is the topic of the review discussed comprehensively in the context of the current literature?

Yes

Reviewer Expertise:

Research funding, research policy

I confirm that I have read this submission and believe that I have an appropriate level of expertise to confirm that it is of an acceptable scientific standard, however I have significant reservations, as outlined above.

# Reviewer report: Reviewer response for version 1

**DOI:** 10.21956/wellcomeopenres.21675.r65759

**Published:** 2023-09-01

**Authors:** Rachel Heyard

**Affiliations:** 1 University of Zurich, Zürich, Switzerland

Stafford and colleagues wrote a comprehensive review on the potential of RCTs to evaluate the effect or the effectiveness of the partial randomisation of funding as an intervention. 

 In the past, many of the decisions done by funders have indeed only partly been backed-up by evidence in the sense that funding allocation processes have been changed without a properly planned experiment to test whether the intervention actually worked. Often it is also not clear what the intervention was supposed to change in the first place. This is mainly because of this experimentation culture the authors refer too is quite new in research funding. Therefore I believe reviews as this one are extremely valuable to introduce the topic to funders. As such, I think it would be crucial to highlight a couple more examples of experiments that have already been done. The authors do refer to The Experimental Research Funder's Handbook in the abstract, but could cite more of the current research on the funding evaluation process. In the end, partial randomisation is an intervention to improve specific aspects of the process so any type of intervention study in research funding could be used as example.   

 More specifically, I have the following comments: 

- [p3 right] In my opinion, the very first step before even deciding on the estimand or outcome of the study is to define a clear and narrow research question, maybe even following the PICOT guidelines. Having a clear research question, will help find the best suited outcome and analysis. (see Riva *et al.*, 2012 ^1^).
- [p4...] Since the authors mention that target outcomes etc should be prespecified, I thought they should also mention (pre)registration of study protocols as it is f.ex. mandatory in clinical trials (to reduce QRPs and other biases). As the authors are suggesting to do more proper experiments and studies in research funding, they should also explicitly highlight best practices in planning such experiments to ensure robustness, reproducibility and transparency.
- [p4] The most suitable outcome should align with the goal of the studied funding scheme or the strategy of the funder. Could something like this be added? 
- [regarding outcome reward and similar] There is no universally agreed on measure of research or societal impact yet - could this be added in the discussion. 
- [sample size of the indicative protocol] The first sentence in the paragraph indicates a clear direction of effect that will be tested - an increase in the total patient benefit. Hence, you would not use a two-sided but a one-sided test (usually with a 2.5%-level) which leads to a sample size of 156 studies per arm. Could you also say something about how one would estimate the target effect (here 0.4)?
- [regarding natural experiments] The effect estimates from regression discontinuity designs (RDD) are unfortunately only valid for applications around the threshold. Plus this threshold is not constant and depends on outside factors such as the number of applications to a call, the available budget, …(see de la Cuesta & Imai, 2016 ^2^).
- [regarding shadow experiments] Could you add some citations of shadow-like experiments that have been conducted in research allocation? (see maybe Graves *et al., *2011 ^3^ or Bieri et al., 2021 ^4^).
- [Conclusion] To not overload funding agencies who might not have the resources nor the expertise to run such experiments by themselves with more tasks it is important to (1) harmonise data among funders and then (2) share it with or collaborate with meta-researchers to get to the best possible outcome. In general it is important to improve transparency of funders when pursuing experiments and involve researchers and panel members/reviewers (i.e. the subjects of study) whenever possible to improve the trust and relationship between funders and the research community. 

Is the review written in accessible language?

Yes

Are all factual statements correct and adequately supported by citations?

Partly

Are the conclusions drawn appropriate in the context of the current research literature?

Yes

Is the topic of the review discussed comprehensively in the context of the current literature?

Yes

Reviewer Expertise:

Statistics, meta-research, funding evaluation

I confirm that I have read this submission and believe that I have an appropriate level of expertise to confirm that it is of an acceptable scientific standard.

## References

[1] : What is your research question? An introduction to the PICOT format for clinicians. *J Can Chiropr Assoc* .2012;56(3) :167-71 22997465 PMC3430448

[2] : Misunderstandings About the Regression Discontinuity Design in the Study of Close Elections. *Annual Review of Political Science* .2016;19(1) : 10.1146/annurev-polisci-032015-010115 375-396 10.1146/annurev-polisci-032015-010115

[3] : Funding grant proposals for scientific research: retrospective analysis of scores by members of grant review panel. *BMJ* .2011;343: 10.1136/bmj.d4797 d4797 10.1136/bmj.d4797 21951756 PMC3181233

[4] : Face-to-face panel meetings versus remote evaluation of fellowship applications: simulation study at the Swiss National Science Foundation. *BMJ Open* .2021;11(5) : 10.1136/bmjopen-2020-047386 e047386 10.1136/bmjopen-2020-047386 33952554 PMC8103360

# Reviewer report: Reviewer response for version 1

**DOI:** 10.21956/wellcomeopenres.21675.r64331

**Published:** 2023-08-17

**Authors:** Becky Ioppolo

**Affiliations:** 1 University of Cambridge, Cambridge, England, UK

Thank you for the opportunity to review this interesting manuscript, which was a pleasure to review. The authors explain that while there is currently a lot of interest in determining research funding by partial randomisation, gathering robust evidence as to whether partial lottery is “better” than other selection strategies would be costly, time-consuming, or otherwise infeasible. Therefore, alternatives to the most robust form of testing (RCTs) are proposed and discussed.

 The manuscript is clear with a logical flow between arguments. The authors clearly outline the necessary planning considerations for funders and metascientists interested in testing or evaluating the possible success of a new decision mechanism for funding. The inclusion of the example of potential partial randomisation in the HTA scheme was particularly illuminating for the challenges that would lie ahead.

 A few opportunities where I would like to suggest amendment, clarification or possible addition:

1. “Steady as she goes” section: Are there no citations for the concerns about small scale studies not aggregating to a compelling evidence base?
2. Table 1: Are there not more citations for the concerns outlined here? Maybe there aren't, but the final two harm outcomes (reputational damage to funder & stigmatisation of awardee) might be mentioned in Liu *et al.* 2020, for example, or similar papers.
3. Table 1: Could you define or illustrate what stigmatisation of awardee would look like? Do you mean specifically the risk that awardees in partial randomisation could be viewed as “not worthy” of funding by peers, or other forms of non-acceptance by scientific community?
4. Table 1: Would it improve clarity in the table if you specified that the harm outcome is “ **Low **quality application”? I assume the intent is that the items in the harm category are all legible as “Potential increase in”
5. Perhaps Table 1 would be more informative to readers if it also included the stated intention(s) of the funders who have undertaken partial randomisation schemes of funders to date (where available).
6. In the final bullet point in the Logistics section of the HTA example, perhaps it is worth adding some clarification such as: e *.g., if an application is rejected, was it rejected at minimum quality threshold stage, at lottery, or via standard procedure committee adjudication?*
7. In the sample size section of the HTA example, it would be great if you could mention how many applicants typically apply for this scheme, which would give readers a sense of how feasible this is.
8. In the feasibility section of the HTA example, should it not be “( **applications** * number of treatment arms)”? In the example you have laid out, applicants have not been mentioned so I’m wondering whether there is a typo in the equation or an omission from a previous section.

There are a few possible typos that may be worth correcting:

1. Introduction – “subset of applications are” should be “subset of applications is”: “In its partial form, only a subset of applications **is **subject to random selection, once those which are evaluated as clearly fundable or clearly non-fundable have been removed.”
2. Considerations... – add comma after e.g.: ( **e.g. ,** schemes limited to early career researchers in a specific area of biomedical science)
3. Table 1 – remove **]** in “Efficiency: Time to deliberation” row.
4. Considerations… - remove comma after hyphen: “…would agree is the most **important - and** a plausible operationalisation…”
5. Considerations… - add comma after e.g.: “( **e.g. ,** partial randomisation and funder standard practice) which would be worth detecting if it existed.”
6. Considerations… - Rephrase: “Outcome: **Patient** benefit arising from both portfolios, calculated by the number **of patients** treated with the HTA-approved products”
7. Alternative to RCTs – add accent for author’s name: “For example **, Hernán** & Robins ( 2016, see also Hernán *et al.*, 2016) propose guidelines for causal inference from large observational databases.”

Is the review written in accessible language?

Yes

Are all factual statements correct and adequately supported by citations?

Partly

Are the conclusions drawn appropriate in the context of the current research literature?

Yes

Is the topic of the review discussed comprehensively in the context of the current literature?

Yes

Reviewer Expertise:

metaresearch, research policy, resarch funding, research evaluation

I confirm that I have read this submission and believe that I have an appropriate level of expertise to confirm that it is of an acceptable scientific standard.
